# Plant Biomechanics—A Natural Transition from Molecular to Organ Scale

**DOI:** 10.3390/ijms23147575

**Published:** 2022-07-08

**Authors:** Dorota Kwiatkowska, Agata Burian

**Affiliations:** Institute of Biology, Biotechnology and Environmental Protection, Faculty of Natural Sciences, University of Silesia in Katowice, Jagiellońska 28, 40-032 Katowice, Poland; agata.burian@us.edu.pl

Plants are multicellular organisms of a unique structure because their tissues consist of two interwoven networks: a network of interconnected protoplasts that is embedded in a network of tightly joined cell walls. Such a structure has significant implications from both developmental and biomechanical perspectives. First, it facilitates mechanical signaling and integration at the organ level. Second, plant tissues can be regarded as cellular solids [1]. An emerging mechanical property of such “constructed” plant organs is that they are prestressed. In the case of green plant organs, built mainly of living tissues, the cellular solids are pressurized by turgid protoplasts. Because tissues within the organ differ in cell size and cell wall stiffness, this pressurization leads to different stresses in different organ portions. Woody stems of living trees are also prestressed. Although they are built mainly of dead cells, the prestress develops during their differentiation. Such natural prestressed constructions are analogous to prestressed concrete, which revolutionized architecture, e.g., the construction of bridges. Also, in the case of plant organs, the prestress, referred to as tissue stress or tensegrity at the organ scale, improves their mechanical performance [2,3]. However, another consequence of such a plant construction is that biomechanical processes acting at subcellular, cellular, and organ scales are closely related and hard to separate, while investigations of molecular mechanisms, in which mechanical factors are involved, are by rule blended with tissue and organ level research.

Although it is obvious that plant bodies of both tiny herbs and huge trees are physical objects and observe rules of mechanics, for decades, mechanical aspects of plant biology have been neglected in a maze of chemical, genetic and molecular details. However, today, the role of mechanics in plant biology is recognized and better understood, thanks to research on the interface between biology, physics, mathematics, and computer science. In accordance with this trend, plant biomechanics has also been introduced to journals focused mainly on molecular biology, such as the *International Journal of Molecular Sciences*. We are thus delighted to introduce this Special Issue of *International Journal of Molecular Sciences*, dedicated to plant biomechanics. It is composed of 10 peer-reviewed papers, 8 articles and 2 reviews, which cover a range of research topics in the field, from the role of mechanical factors in regulation of plant development, to the mechanical design of the plant body that facilitates specific function performance and adaptation to environment.

How plants sense, transduce, and respond to mechanical signals are fundamental questions in plant development. In their comprehensive review, Hartmann et al. [4] discuss the role of mechanosensitive calcium channels in conversion of mechanical signals into chemical ones. Their detailed literature review is complemented with data mining and a novel visualization approach. Using this approach, the authors identify relationships between the location of mechanosensitive channels, the localization of stress, and stress responses in primary tissues of the annual herb, *Arabidopsis thaliana*, and differentiating secondary wood tissues of the dicot tree, aspen (*Populus tremula*). They discuss in detail the molecular aspects of the mechanosensing mechanism in plant kingdom on the background of an overall tree of life. In conclusions, Hartmann et al. [4] point to fundamental and challenging questions on the putative relationships between activation of the mechanosensitive channels and membrane tension, as well as the possible transcriptional regulation of channel building proteins.

Saikia et al. [5] focused on kinematic aspects of mechanotransduction, in which mechanosensitive ion channels are involved. They studied sensory hairs of Venus flytrap (*Dionaea muscipula*), which sense touching of trap lobe surfaces by insects, which eventually leads to sudden trap closure. Saikia et al. [5] combined cutting-edge empirical analyses of structure and mechanics of sensory hairs, with modelling of hair deformation at the tissue and subcellular scales. The comprehensive 3D multiscale model, based on the finite element method (FEM), in which detailed empirical data were used as the input, allowed them to calculate local stretch of individual cell walls of the hair. This stretch takes place when the hair is deflected due to the touch stimulus. Surprisingly, the computed location of the highest stress in cell walls was counterintuitive. Such highly stretched portions of cell walls likely define preferable locations of mechanosensitive ion channels in the adjacent plasma membrane, providing mechanotransduction “hotspots”. Furthermore, Saikia et al. [5] performed simulations for hairs of different shapes and showed the significant relation between cell and tissue geometry and the hotspot location.

The geometry of both cells and tissues is also significant for stiffness of growing plant tissues. Walls of cells in the growing tissues need the ability to expand with the specific anisotropy (e.g., elongate along the organ axis) while being stiff enough to resist turgor (the internal hydrostatic pressure exerted by protoplast). Majda et al. [6] combined empirical experiments using micro-extensometer and simulation “experiments” using original FEM-based models, in order to explain counterintuitive observations on adaxial (inner) epidermis of young bulbs of onion (*Allium cepa*). This easily separable tissue comprises a single layer of elongated cells. Surprisingly, extensometer measurements on the living onion epidermis show that it is stiffer along the cell axis, which is the direction of their maximal growth, than in the transverse, i.e., minimal growth direction. Performing numerous simulations accounting for various real or idealized cell patterns, and mechanical properties, Majda et al. [6] elucidate complex relationships between stiffness of the turgid tissue and its cellular organization, which explain the above mentioned contradiction.

The surface of the epidermis of plant shoots is covered by a layer of waterproof cuticles. Skrzydeł et al. [7] discuss mechanical aspects of structure, assembly, and function of cuticles. The focus of this review is the cuticle of perianth organs, i.e., sepals, petals or tepals, which is special in both its structure and function. These peculiarities are often related to the cuticle mechanics, such as in the case of a folded cuticle that functionally resembles profiled plates, or the petal epidermis of some plants, where the cuticle is the only continuous layer on the organ surface. Skrzydel et al. [7] point to gaps in our knowledge on formation of cuticle patterns and their role in perianth organ function.

Two original articles of this Special Issue are dedicated to relations between the mechanical properties of cells or tissues and the chemical composition and structure of cell walls. Modifications of composition and structure of cell walls may affect commercial quality of plant crops. Castro et al. [8] studied the role of auxin in softening of strawberry (*Fragaria* x *ananassa*) fruit (from a developmental perspective, strawberry is the enlarged receptacle with the attached achenes, which are true fruits of this crop plant [9]). In the final stage of strawberry fruit ripening, the cell wall disassembly takes place, in the regulation of which auxin is involved. The process is significant for the commercial crop value. Castro et al. [8] performed physicochemical analyses of cell walls of fruit tissues during this softening process. They also combined biochemical and molecular biology tools with fruit quality analyses related to fruit mechanical properties, which are influenced by cell wall remodeling. The authors were able to show the influence of auxin treatment on integrity and stability of polysaccharides of the fruit cell walls using a novel method of thermogravimetric analysis of plant tissues.

Some plant cells produce extracellular mucilage. It is a complex of differently structured polysaccharides, such as hemicellulose and pectin, and may also contain cellulose. Mucilage components are thus similar to those of growing cell walls. Kreitschitz et al. [10] studied mucilage-mediated adhesion of plant seeds to artificial media. They evaluated adhesive properties for mucilage produced by seeds of six plant species, which are to various extent related, and complemented these measurements with literature data on mucilage structure and composition. The adhesion strength of dried seed mucilage is generally high, and related to its polysaccharide structure, which varies between mucilage produced by seeds of different plant species. Seed adhesion by mucilage to transporting agents (e.g., animals) may help in their transport, while adhesion to the ground may increase the survival rate of seeds. Thus, the results of this study are important not only for plant biomechanics but also from the perspective of ecology and seed biology.

Extracellular material plays a different function in the case of grass roots. Potocka and Szymanowska-Pułka [11] studied a pellicle, which is an often overlooked deposit of extracellular material covering root apices of grasses. Their original experimental setup, in which mechanical stress was applied to growing root tips of maize (*Zea mays*), combined with comprehensive structural studies, allowed the authors to recognize how stress influences the pellicle structure and viability of root cells. They showed that in response to mechanical stress, pellicle structure changes, while its thickness increases. Interestingly, even though the stress causes pellicle microdamage, the meristematic tissue at the root tip remains intact. Thus, Potocka and Szymanowska-Pułka [11] postulate that in grasses, pellicle is crucial for protection of meristematic root tissues from mechanical stress.

Protective functions are performed by a wide range of organ “coatings”, from an extracellular material mentioned above to bark covering woody stems, which is a complex of various tissues. Bold et al. [12] studied the mechanical behavior of the bark of giant sequoia (*Sequoiadendron giganteum*). This bark is a layered hierarchical structure. It provides protection against rockfall events by dissipation of energy from the high-energy impacts, which would otherwise damage cambium, the meristem responsible for secondary growth in thickness of woody stems. Bold et al. [12] performed detailed anatomical analyses and comprehensive mechanical tests at the scales of cell (single fiber), tissue, and tissue complex (whole bark). The results allowed them to explain the mechanism of energy dissipation by the giant sequoia bark, which is based on its pronounced viscoelastic deformation. Bold et al. [12] discuss this mechanism and various aspects of mechanical performance of giant sequoia bark in comparison to other structures and materials, both biological and technical.

In some plants, outer tissues are involved in a process of self-repair of mechanically damaged shoots. The initial phase of the self-repair is a rapid self-healing, in the course of which the wound is sealed in order to limit the water loss and protect the plant from pathogen invasion. This phase is followed by a long-lasting phase of self-healing, during which the damaged tissues are regenerated and mechanical properties at least partly restored [13]. The mechanism of self-sealing was studied by Hesse et al. [14] for leaves of two succulent plant species from the genus *Delosperma*, adapted to habitats differing in air humidity and water availability. After comprehensive comparison of leaf morphology and anatomy, as well as mechanical properties of leaves and leaf tissues of the two *Delosperma* species, Hesse et al. [14] followed in detail the kinematics of the self-sealing process after a ring incision. The self-sealing process of *Delosperma* leaves is accomplished by uneven leaf contraction, which brings the wound edges into contact. Hesse et al. [14] showed that a significantly higher leaf contraction in the species adapted to more arid regions is related to specific elastic properties of its vascular tissues.

The self-repair process in cacti branches was studied by Mylo et al. [13]. They performed a comprehensive morphological and anatomical analysis of this process in two closely related cacti species from genera *Opuntia* and *Cylindropuntia*. This analysis was combined with series of measurements of mechanical properties of wounded branches, up to the completion of the self-repair process. The obtained results allowed them to compare the effects of the restoration of structural integrity, which takes place during the self-repair, on the water-retaining and mechanical properties of the wounded branches. They postulate that in the examined cacti, protection against water loss and pathogen infection after wounding is under higher selection pressure than a complete restoration of mechanical performance. Mylo et al. [13] also point to an interesting analogy between the wound healing and an abscission process, which facilitates fruit shedding or vegetative propagation by branch separation.

Important outcomes of the studies presented in this Special Issue are the original models [5,6] and tools for mechanical measurements adapted to plant material, such as micro-extensometers [6,12] and devices developed for the measurement of adhesive force [10] or bending stiffness [13,14]. It is also noteworthy that the presented results are often beneficial for applied science. One article is directly related to food quality [8]. In other articles, the possible applications in agriculture, material science or biomimetics are referred to. Hartmann et al. [4] discuss practical applications of our knowledge on mechanosensing and mechanotransduction using an example of thigmomorphogenesis. The results of the investigations on the Venus flytrap sensory hair [5] can become beneficial for the design of bio-inspired sensors. Physical and chemical characteristics of natural seed mucilage [10] may inspire the production of nontoxic water-based glues. The specific structure of giant sequoia bark, responsible for its capability of energy dissipation, is relevant for application in civil engineering [12]. Finally, the natural process of self-repair studied by Hesse et al. [14] and Mylo et al. [13] can be inspiring for the invention of self-repairing material systems.

Overall, the present Special Issue highlights diverse aspects of plant biomechanics and offers an inspiring overview of key research topics. As the Guest Editors, we wish to warmly thank all the authors for their significant, interesting, and inspiring contributions to this paper collection. We also thank the *International Journal of Molecular Science* for the support.
